# Severe burn injury alters intestinal microbiota composition and impairs intestinal barrier in mice

**DOI:** 10.1186/s41038-019-0156-1

**Published:** 2019-07-04

**Authors:** Yanhai Feng, Yalan Huang, Yu Wang, Pei Wang, Fengjun Wang

**Affiliations:** 1 State Key Laboratory of Trauma, Burns, and Combined Injury, Institute of Burn Research, Southwest Hospital, Third Military Medical University (Army Medical University), 30 Gaotanyan Street, Chongqing, 400038 China; 2 Department of Military Nursing, School of Nursing, Third Military Medical University (Army Medical University), Chongqing, China; 3 Department of Gastroenterology, Southwest Hospital, Third Military Medical University (Army Medical University), Chongqing, China

**Keywords:** Burn, Intestinal barrier, Tight junction protein, Intestinal microbiota, Short-chain fatty acids, Inflammatory cytokines

## Abstract

**Background:**

The intestinal barrier integrity is crucial for maintaining intestinal homeostasis, and the mechanisms of intestinal barrier disruption induced by burn injury remain obscure. This study was aimed to investigate the changes of intestinal microbiota and barrier function in burned mice to further comprehend the mechanisms of burn-induced intestinal barrier dysfunction.

**Methods:**

Samples were from mice inflicted with 30% total body surface area (TBSA) full-thickness burns. The intestinal permeability, tight junction proteins expressions, zonula occludens-1 (ZO-1) localization, inflammatory cytokines expressions, and short-chain fatty acids (SCFAs) contents were determined. The microbial community was assessed via 16S rDNA Illumina sequencing.

**Results:**

The intestinal permeability was increased after severe burn injury, peaking at 6 h post-burn, with approximately 20-folds of the control (*p* < 0.001). The expression of tight junction proteins (ZO-1, occludin, claudin-1, and claudin-2) was significantly altered (*p* < 0.05). The ZO-1 morphology was dramatically changed following burn injury. The fecal SCFAs’ contents (acetate, propionate, butyrate, isobutyrate, and isovalerate) were noticeably declined after burn injury (*p* < 0.05). The expressions of pro-inflammatory cytokines (interleukin (IL)-1β and IL-6) in ileal mucosa were increased, whereas the expressions of anti-inflammatory cytokines (IL-4 and IL-13) were decreased following burn injury (*p* < 0.05). In addition, burned mice showed an alteration of intestinal microbial community, such as decreased diversity, reduced *Bacteroidetes* abundance, and increased *Firmicutes* abundance.

**Conclusions:**

The severe burn-induced intestinal barrier dysfunction is along with the alterations of microbial community.

**Electronic supplementary material:**

The online version of this article (10.1186/s41038-019-0156-1) contains supplementary material, which is available to authorized users.

## Background

The intestinal epithelial barrier integrity plays a crucial role in preventing pathogens and other molecules from entering the intestinal mucosa and interacting with the immune system, in which tight junction are principal structural factors [1–4]. However, the intestinal barrier is destroyed in some severe surgical diseases, such as trauma, shock, and burn injury, resulting in augmented intestinal permeability and subsequent bacterial translocation or/and endotoxin. The disrupted intestinal epithelial barrier is responsible for systemic inflammatory response syndrome, sepsis, multiple organ dysfunction syndrome, and other severe complications [5–7]. Thus, further investigating the mechanisms of intestinal barrier disruption and developing corresponding therapeutic strategies to maintain intestinal barrier are meaningful.

Virtually, human individuals live in close association with surrounding microbes, especially the microorganisms inhabiting in the gastrointestinal tract. The microorganisms residing in the gastrointestinal tract are referred as the gut microbiota [8], which is important for host physiological and pathological processes such as intestinal epithelial barrier reinforcement, immune system development, and nutrients absorption [9]. It has been demonstrated that intestinal ischemia/reperfusion, which often occurs at the early stage of severe burn injury, induce the dysbiosis of gut microbiota [10]. However, the detailed alteration of intestinal microbiota composition in severe burn injury still needs to be clarified.

Short-chain fatty acids (SCFAs), including acetate, propionate, butyrate, isobutyrate, and isovalerate, are bacterial metabolites generated through fermentating dietary fibers. Luminal SCFAs, which are recognized as preferred energy substrates for colonic epithelia, influence epithelial barrier function, mucosal immune systems, and inflammatory responses [11], for example, butyrate for colitis by promoting regulatory T cells’ (Tregs) formation, decreasing pro-inflammatory cytokines, activating Gpr109a to suppress inflammatory signals, and enhancing intestinal barrier [12–17]. It has been documented that gut microbiota deficiency is associated with increased blood-brain barrier permeability, which may be attributed to altered SCFAs [18]. Even so, the role of SCFAs on intestinal barrier remains obscure.

In this study, we report that severe burn injury could disrupt intestinal microbiota community, alter inflammatory cytokines, decrease luminal SCFAs, and disrupt intestinal epithelial barrier. Thus, it is suggested that intestinal microbiota dysbiosis may contribute to the barrier dysfunction following a severe burn.

## Methods

### Animals

Healthy adult female C57BL/6 mice weighing 18–22 g were used in this study. All the mice were supplied by the Animal Institutes of Daping Hospital, the Third Military Medical University (Army Medical University). The animals were housed in wire-bottomed and wire-lid cages, allowed access to chow and water ad libitum, and acclimatized for 1 week before experiments in a temperature-controlled room (25 ± 2 °C) with 12-h light and dark cycles. The animal studies were approved by the Animal Care and Use Committee of Third Military Medical University (Army Medical University), and all the protocols were approved by the Medical and Ethics Committee of Southwest Hospital, Third Military Medical University (Army Medical University), Chongqing, China.

### Burn model and experimental protocol

The burn model was created according to the method usually used in our laboratory [19]. In brief, the control group and burn group received the same treatment expect the scald burn. At the end of the experiment, the mice were anesthetized to detect intestinal permeability and collect intestinal tissue and fecal samples. The control mice were sacrificed immediately after sham burn treatment (0 h), but not at each time point. The harvested tissues and feces were respectively used for immunofluorescent assay, immunoblot, liquid chip, headspace gas chromatography-mass spectrometry (HS-GC/MS), DNA isolation, and microbiota community analysis, as described below.

### *In vivo* intestinal paracellular permeability assay

In this part, we applied the methods which described in previous articles [19]. In brief, after the mice were anesthetized, a 5-cm segment of ileum was dissociated, and then, 0.1 ml of 20 mg/ml fluorescein isothiocyanate-labeled (FITC)-dextran was injected in this segment. After 30 min, the blood sample was collected for fluorescence intensity detection by using a microplate reader (Varioskan Flash, Thermo Electron Corporation, Vantaa, Finland) with wavelength of 480 nm/520 nm. And then, the intestinal permeability was calculated according to the standard curve.

### Immunofluorescent staining, microscopy, and image analysis.

We applied the methods which usually utilized and described in previous articles [19]. Frozen sections of ileum were prepared for detecting ZO-1 morphology. After preparatory work, the sections were incubated with monoclonal rabbit antibody against zonula occludens (ZO)-1 (Invitrogen, Carlsbad, CA) diluted at 1:200 in 5% normal goat serum in phosphate buffer saline (PBS) at 4 °C overnight. Then the sections were incubated with secondary Alexa Fluor 488-conjugated goat anti-rabbit IgG antibody (Invitrogen) at 1:200, Alexa Fluor 594-conjugated phalloidin (Invitrogen) at 1:100, and diamidine-2-penylindole (DAPI) (Sigma) at 1:1000 for 1 h at room temperature. Images were obtained using a TCS SP5 laser confocal microscopy (Leica, Germany).

### Immunoblot analysis of tight junction proteins

In this part, we applied the methods described in previous articles [19]. In brief, the total tissue proteins were collected for the determination of ZO-1, occludin, claudin-1, and claudin-2. By the way, equal amounts of total protein extracted from the ileal mucosa were respectively fractionated on 8%, 10%, and 12% sodium dodecyl sulfate- polyacrylamide gel electrophoresis (SDS-PAGE) gel and then transferred to polyvinylidene difluoride (PDVF) membrane (Millipore, Bedford, MA). Furthermore, after the membrane was blocked, the membranes were incubated with antibodies specific for ZO-1 (1:1000, Invitrogen), occludin (1:1000, Invitrogen), claudin-1 (1:1000, Invitrogen), claudin-2 (1:1000, Abcam), and β-actin (1:5000, Sigma) overnight at 4 °C. After washing with Tris-buffered saline Tween-20 (TBST), membranes were incubated with appropriate peroxidase-conjugated secondary antibodies (KPL, America) at room temperature for 60 min. The chemiluminescence signal was captured using a ChemiDox XRS system (Bio-Rad). The densities of the bands were quantified with Quantity One Image software (Bio-Rad).

### Fecal SCFAs analysis via HS-GC/MS

Fresh ileocecal fecal matters were frozen immediately and stored at − 80 °C until processing. The weighed fecal matters were put into a 20 ml headspace sampling bottle, followed by the addition of 0.95 ml 6% H_3_PO_4_ and 0.05 ml 2.49 mmol/l of 2-ethylbutyric acid (the internal standard, diluted by 6% H_3_PO_4_), and vibration for 1 min. Then, the samples were injected into the GC/MS system (5975A/7890C Agilent) for analysis. Ultrapure Helium (1.0 ml/min) was used as the carrier gas. Headspace was maintained at 80 °C with incubation time of 30 min. The gas chromatography (DB-FFAP fused-silica capillary column: 30 m × 0.25 mm × 0.25 μm Agilent) stepwise thermal conditions were as follows: 50 °C for 1 min, 10 °C/min until 250 °C for 2 min; the injector temperature was 250 °C, with split ratio of 5: 1. The mass detector system was operated in electron impact ionization at 70 eV, set to scan mode for m/z 33–200, and in single ion monitoring (SIM) mode. The temperatures of the transfer line (interface) and source were maintained at 280 °C and 250 °C, respectively. The monitored ions were m/z 60 and 43.1 for acetic acid, m/z 74.1 and 28.1 for propionic acid, m/z 60 and 73.1 for butyric acid, m/z 60 and 87.1 for isovaleric acid, m/z 43.1 and 73.1 for isobutyric acid, and m/z 88.1 and 73.1 for 2-ethylbutyric. Data acquisition and analysis were done with Saturn GC/MS workstation software (Agilent).

### Microbial diversity analysis

#### DNA extraction

Fresh ileocecal fecal matters were collected and frozen immediately at − 80 °C for DNA extraction. Microbial DNA was extracted with the QIAamp® Fast DNA Stool Mini Kit (Qiagen, Valencia, CA) according to the manufacturer’s protocols and then stored at − 80 °C.

#### Polymerase chain reaction (PCR) amplification

The V3-V4 region of bacterial 16S rDNA gene was amplified using primers 338F (5′-ACTCCTACGGGAGGCAGCAG-3′) and 806R (5′-GGACTACHVGGGTWTCTAAT-3′). The PCR reactions (95 °C for 3 min, followed by 27 cycles at 95 °C for 30 s, 55 °C for 30 s, and 72 °C for 45 s and a final extension at 72 °C for 10 min) were performed in 20-μl mixture containing 4 μl of 5× FastPfu Buffer, 2 μl of 2.5 mmol/l dNTPs, 0.8 μl of primers (5 μmol/l), 0.4 μl of FastPfu Polymerase, and 10 ng of template DNA. Illumina MiSeq sequencing amplicons were extracted from 2% agarose gels, purified using the AxyPrep DNA Gel Extraction Kit (Axygen Biosciences, Union City, CA, USA) according to the manufacturer’s instructions, and quantified using QuantiFluor™-ST (Promega, USA). The purified amplicons were pooled in equimolar and paired-end sequenced (2 × 250) on an Illumina MiSeq platform according to the standard protocols. The raw reads were deposited into the NCBI Sequence Read Archive (SRA) database.

#### Processing of sequencing data

The raw fastq files were demultiplexed, quality-filtered using QIIME (version 1.9.1) with the following criteria: (i) the 300 bp reads were truncated at any site receiving an average quality score < 20 over a 50 bp sliding window, discarding the truncated reads that were shorter than 50 bp; (ii) exact barcode matching, 2 nucleotide mismatch in primer matching, reads containing ambiguous characters were removed; (iii) and only sequences that overlap longer than 10 bp were assembled according to their overlap sequence. The reads which could not be assembled were discarded. Operational taxonomic units (OTUs) were clustered with 97% similarity cutoff using UPARSE (version 7.1 http://drive5.com/uparse/), and chimeric sequences were identified and removed using UCHIME. The taxonomy of each 16S rRNA gene sequence was analyzed by RDP Classifier (http://rdp.cme.msu.edu/) against the silva (SSU123)16S rRNA database using confidence threshold of 70% [20].

### Inflammatory cytokines analysis by liquid chip

After the animals were sacrificed, an appropriate ileal segment was taken to harvest mucosa by a glass slide. The harvested mucosa was homogenized in ice-cold PBS containing protease inhibitor cocktails (Millipore), followed by a brief sonication with a sonicator (Tomy Seiko, Tokyo, Japan), and a centrifugation at 12000 rpm, 4 °C for 15 min. The supernatants were collected for the determination of inflammatory cytokines by Merck Millipore Liquid Chip Kit (Millipore) according to the manufacturer’s protocols.

### Statistical analysis

Data are presented as means ± standard error of the mean (SEM). For data analysis, Dunnett’s *t* test was performed against the control group only using SPSS 13.0 statistical software. A *p* value of < 0.05 was considered as minimum level of significance in all cases. All reported significance levels represent two-tailed *p* values.

## Results

### Severe burn increased intestinal permeability

**Fig. 1:**
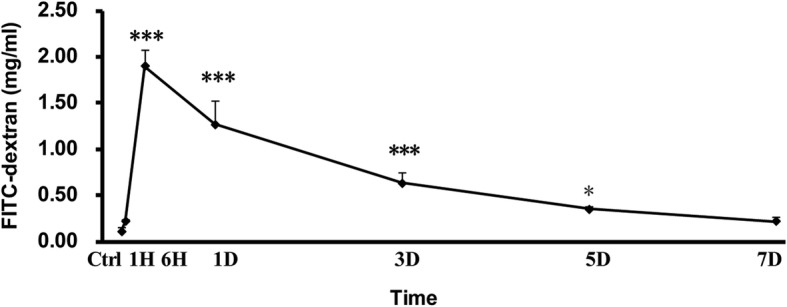
Severe burn increased intestinal permeability. Intestinal permeability was assessed by measuring fluorescein isothiocyanate-labeled (FITC)-dextran concentration in the systemic circulation after intraluminal injection of 4 kDa FITC-dextran at the indicated time points after 30% total body surface area (TBSA) burn injury. Intestinal permeability to 4 kDa FITC-dextran was significantly increased after burn injury. Data represent the mean ± standard error of the mean (SEM) (*n* = 5–6). *p < 0.05, ***p < 0.001 compared with 0 h (control), analyzed using Dunnett's *t* test. Ctrl stands for control group, 1H, 6H stands for 1-h, 6-h post-burn group, 1D, 3D, 5D, 7D stands for 1-day, 3-day, 5-day, 7-day post-burn group

We analyzed the intestinal barrier permeability in mice (sham or 30% total body surface area (TBSA) full-thickness burn) through measuring the concentration in plasma of 4.4 kDa FITC-dextran, which is traditionally used in this experiment. As shown in Fig. 1, when compared with control (0 h), the FITC-dextran concentration started to increase significantly at 1 h after burn, peaked at 6 h with approximately 20-folds of control, and was still significantly higher than that of control on 1, 3, 5, and 7 days after burn injury. These results indicate that the intestinal paracellular permeability is significantly increased in burned mice, suggesting that severe burn injury can destroy intestinal epithelial barrier.

### Severe burn changed tight junction proteins’ expressions

**Fig. 2:**
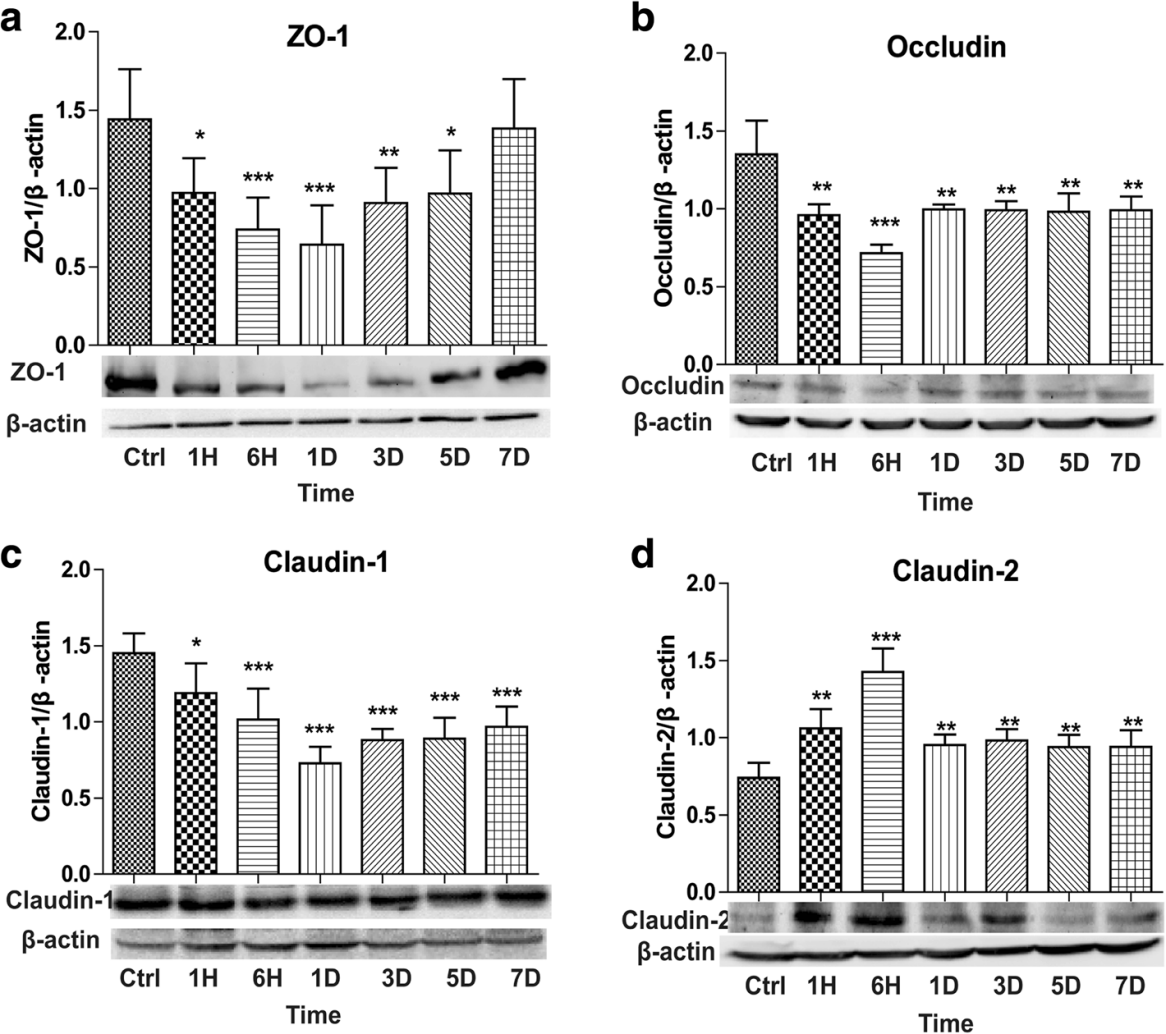
Severe burn changed tight junction proteins’ expressions. The expressions of tight junction proteins were assessed by Western blot after 30% total body surface area (TBSA) burn injury. **a**–**c** Burn injury significantly decreased the protein expressions of zonula occludens (ZO)-1, occludin, and claudin-1. **d** Burn injury significantly increased the expression of claudin-2 protein. Data represent the mean ± standard error of the mean (SEM) (occludin, *n* = 6; ZO-1, claudin-1, claudin-2, *n* = 5 ), *p < 0.05, **p < 0.01, ***p < 0.001 as compared with control, analyzed using Dunnett's *t *test. Ctrl stands for control group, 1H, 6H stands for 1-h, 6-h post-burn group 1D, 3D, 5D, 7D stands for 1-day, 3-day, 5-day, 7-day post-burn group

To more precisely understand the intestinal barrier dysfunction induced by burn injury, we next evaluated the expressions of ZO-1, occludin, claudin-1, and claudin-2, which are important members of tight junction proteins. As illustrated in Fig. 2a–c, when compared with control, the expressions of ZO-1, occludin, and claudin-1 decreased significantly after burn injury. Both ZO-1 and claudin-1 reduced to the minimum on 1 day post-burn, whereas occludin dropped to the lowest level at 6 h post-burn. In addition, ZO-1 expression gradually increased after 1 day and almost increased to the control level on 7 days post-burn. The expressions of both occludin and claudin-1 following burn injury were always lower than those of control. On the contrary, as shown in Fig. 2d, claudin-2, a pore-forming protein [21], was remarkably increased at all time points after burn injury, peaking at 6 h post-burn. These results indicate that the expressions of tight junction proteins are significantly changed after severe burn injury, which may result in the disruption of intestinal barrier.

### Severe burn disrupted ZO-1 morphology

**Fig. 3:**
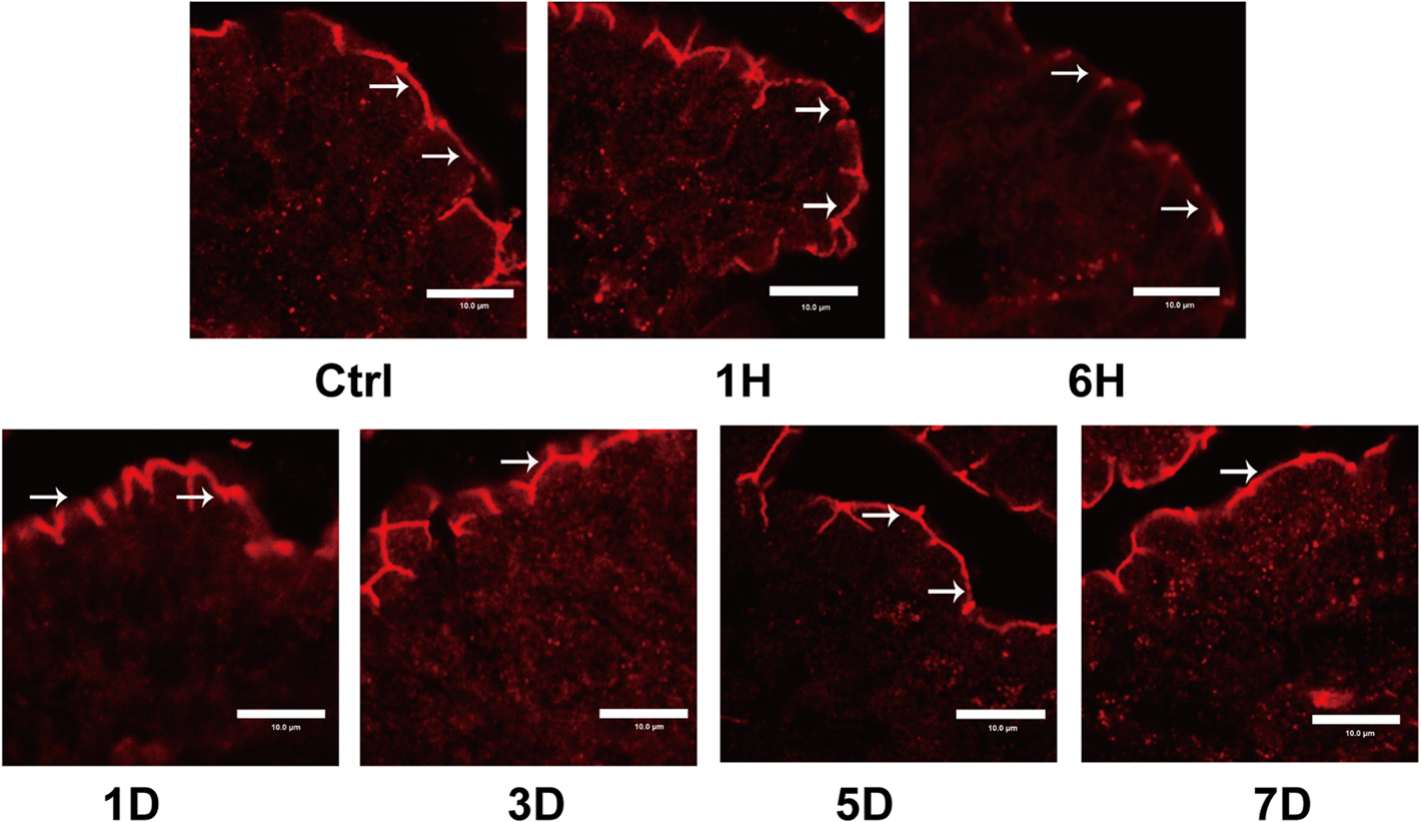
Severe burn disrupted zonula occludens (ZO)-1 morphology. Frozen sections of distal ileum were labeled for ZO-1 (red) by immunofluorescent staining. ZO-1 was localized to the epithelial tight junctions in the ileum of control mice. In burned mice, ZO-1 was stained at the apical junctions, but accompanied by the relocalization of ZO-1. Scale bar = 10.0 μm. Ctrl stands for control group, 1H, 6H stands for 1-h, 6-h post-burn group, 1D, 3D, 5D, 7D stands for 1-day, 3-day, 5-day, 7-day post-burn group

To more comprehensively illuminate severe burn-induced intestinal barrier deficiency, we assessed the alterations of ZO-1 morphology by immunofluorescent. As shown in Fig. 3, in control mice, ZO-1 was distributed to the epithelial tight junction, which was appreciated as red spots and almost a straight line, lying at the apical compartment of cell-cell junctions. In contrast, the ordered ZO-1 morphology was disrupted in the ileum of burned mice, especially at 6 h post-burn. These morphological alterations of ZO-1 were consistent with the abovementioned changes of intestinal permeability after burn injury. Thus, severe burn injury could alter ZO-1 morphology, contributing to the intestinal barrier dysfunction.

### Severe burn changed inflammatory cytokines’ expressions

**Fig. 4:**
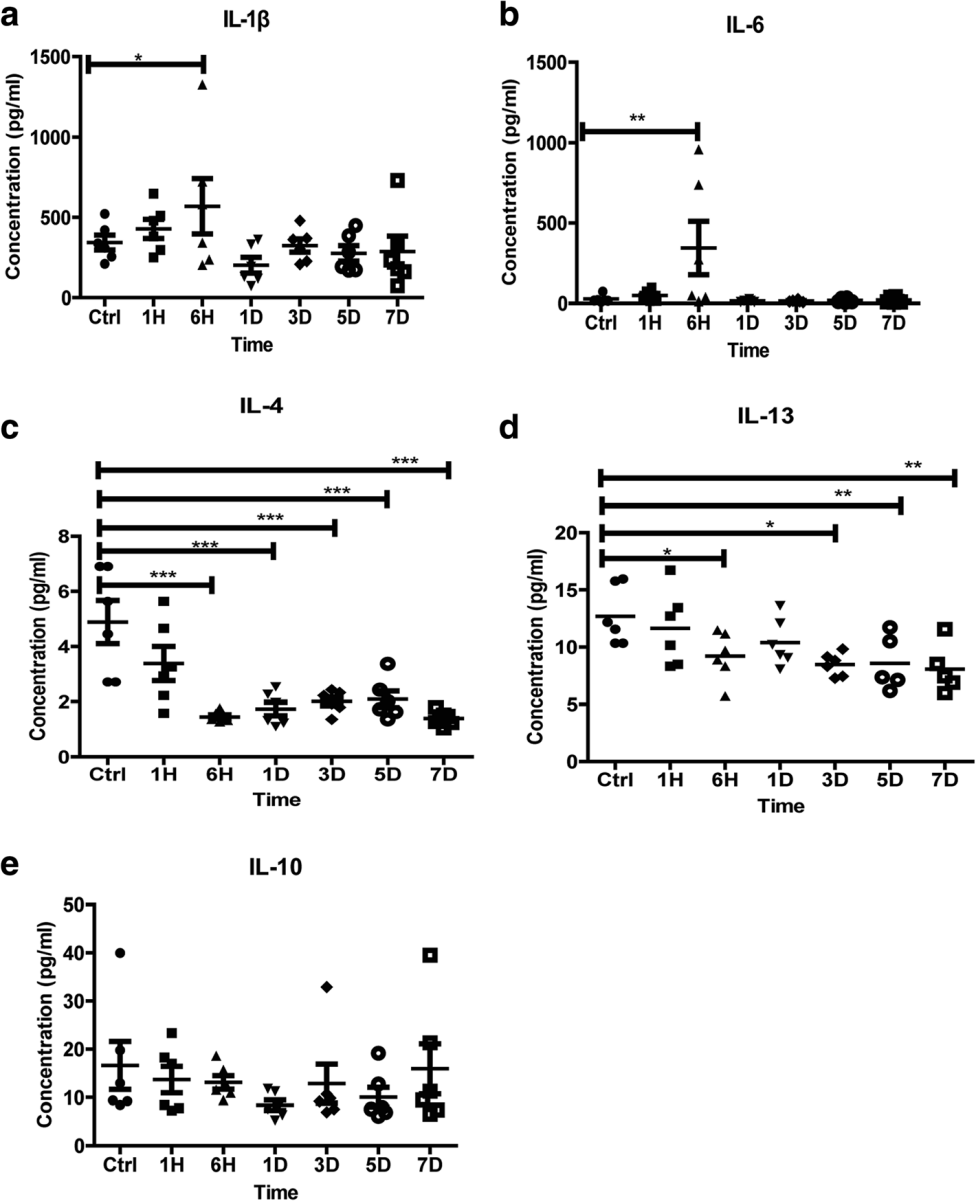
Severe burn changed inflammatory cytokines’ expressions. The expressions of inflammatory cytokines (interleukin (IL)-1β, IL-6, IL-4, and IL-13) in ileal mucosa were assessed after 30% tatal body surface area (TBSA) burn injury. Burn injury significantly increased the contents of pro-inflammatory cytokines IL-1β (**a**) and IL-6 (**b**) and remarkably decreased the contents of anti-inflammatory cytokines IL-4 (**c**), IL-13 (**d**) and IL-10 (**e**). Data represent the mean ± standard error of the mean (SEM) (*n*=6), *p < 0.05, **p < 0.01, ***p < 0.001 compared with control, analyzed using Dunnett's* t* test. Ctrl stands for control group, 1H, 6H stands for 1-h, 6-h post-burn group, 1D, 3D, 5D, 7D stands for 1-day, 3-day, 5-day, 7-day post-burn group

To determine the involvements of inflammatory cytokines in the burn-induced intestinal barrier dysfunction, we analyzed the expressions of interleukin (IL)-1β, IL-6, IL-4, IL-10, and IL-13 in ileal mucosa. As shown in Fig. 4, the contents of pro-inflammatory cytokines IL-1β and IL-6 in ileal mucosa of burned mice were much higher than those of control mice, especially at 6 h post-burn (Fig. 4a, b). However, the contents of anti-inflammatory cytokines IL-4 and IL-13 in ileal mucosa of burned mice were dramatically lower than those of control mice (Fig. 4c, d). Importantly, anti-inflammatory cytokine IL-10 showed slight alteration without statistical significance. These results indicate that severe burn could motivate the inflammation in intestinal mucosa, which may be the prominent contributor of intestinal barrier impairment in the early stage of severe burn injury.

### Severe burn decreased fecal SCFAs contents

**Fig. 5:**
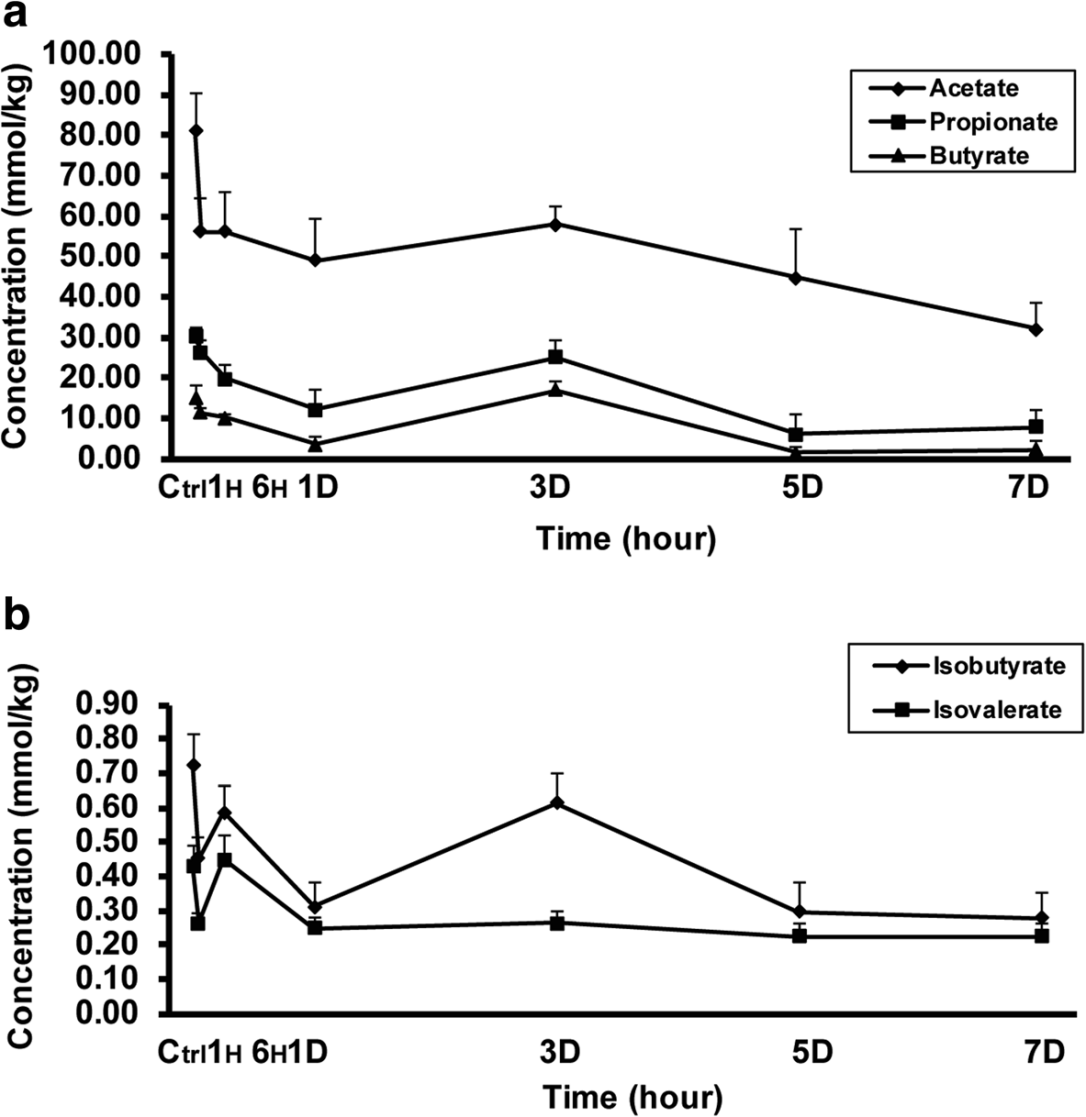
Severe burn decreased fecal contents of short-chain fatty acids (SCFAs). The contents of SCFAs (acetate, propionate, butyrate, isobutyrate, and isovalerate) in ileocecal feces were assessed after 30% total body surface area (TBSA) burn injury. Burn injury remarkably decreased the contents of all SCFAs. Data represent the mean ± standard error of the mean (SEM) (*n*=6). Ctrl stands for control group, 1H, 6H stands for 1-h, 6-h post-burn group, 1D, 3D, 5D, 7D stands for 1-day, 3-day, 5-day, 7-day post-burn group

It has been demonstrated that SCFAs act not only as the dominant energy resources of intestinal epithelia but also as the anti-inflammatory factors [11]. Therefore, to more precisely illuminate the underlying mechanisms of intestinal barrier dysfunction induced by severe burn injury, we next investigated whether severe burn injury was accompanied by the alteration of fecal SCFAs contents. Table 1 and Fig. 5 demonstrated the concrete contents and variation tendencies respectively. The contents of all SCFAs, including acetate, propionate, butyrate, isobutyrate, and isovalerate, decreased gradually from 1 h to 7 days post-burn, and dropped to the minimum on 7 days. Thus, severe burn injury could statistically downregulate the contents of intestinal SCFAs, which might contribute to the intestinal inflammation and barrier dysfunction.

**Table 1:** Fecal contents of short-chain fatty acids (SCFAs) (mmol/kg, *n* = 5 or 6) in burned mice

SCFAs	Groups						
	Control	1-hour	6-hour	1-day	3-day	5-day	7-day
Acetate	81.11 ± 9.25	56.25 ± 8.33	56.19 ± 9.92	49.16 ± 10.24*	58.08 ± 4.45	44.85 ± 11.91*	32.11 ± 6.40***
Propionate	30.25 ± 1.96	26.51 ± 3.10	19.97 ± 3.50*	12.14 ± 5.17**	25.17 ± 4.13	6.17 ± 4.82***	7.81 ± 4.28**
Isobutyrate	0.72 ± 0.09	0.45 ± 0.06*	0.59 ± 0.08	0.31 ± 0.07**	0.61 ± 0.08	0.30 ± 0.08**	0.28 ± 0.07**
Butyrate	15.29 ± 2.95	11.41 ± 1.45	10.30 ± 0.71	3.53 ± 1.91**	17.01 ± 2.22	1.65 ± 1.29**	2.26 ± 1.98**
Isovalerate	0.43 ± 0.06	0.26 ± 0.03*	0.45 ± 0.07	0.25 ± 0.03*	0.27 ± 0.03	0.23 ± 0.03*	0.22 ± 0.04*

Values represent the mean ± SEM. *SEM *standard error of the mean

*p < 0.05, **p < 0.01, ***p < 0.001 when compared with control, Dunnett's *t *test was used for analysis

### Sequencing coverage and measurement of bacterial diversity

It has been illuminated that SCFAs are metabolites of intestinal microbiota [11]. To further identify the mechanisms of severe burn-induced intestinal barrier dysfunction, we next evaluated whether severe burn injury could influence intestinal microbiota composition utilizing an Illumina high-throughput sequencing technique. We created a dataset which is composed of 1,443,620 filtered high-quality and classifiable 16S rDNA gene sequences. The average sequences number of each sample was 36,090.5 (range from 30,093 to 44,839). All sequences were clustered with representative sequences, and a 97% sequence identification cutoff was used. The obtained total number of operational taxonomic units (OTUs) was 11,878. For each sample, bacterial species richness leveled off as sampling depth increased (Additional file 1: Figure S1), indicating that the sequencing effort was sufficiently and that the total diversity within the samples was captured. As shown in Table 2, the microbial richness decreased significantly on 5 days post-burn as compared with the control (*p* = 0.034). A diversity analysis based on Shannon and Chao [22] indexes revealed that the diversity of the intestinal microbiota at the OTU level was significantly lower than that of control at 1 h (*p* = 0.037), 5 days (*p* = 0.031), and 7 days (*p* = 0.036) post-burn. These results confirm that severe burn injury remarkably decreases the diversity and richness of intestinal microbiota.

**Table 2:** Sequencing results of observed operational taxonomic units (OTUs) and diversity indices

Parameters	Control	1-hour	6-hour	1-day	3-day	5-day	7-day
OTU	317.5 ± 14.85	288 ± 15.62	317.83 ± 3.54	319.33 ± 6.02	285.67 ± 11.75	259 ± 13	278.67 ± 10.95
Chao [22]	356.5 ± 16.45	335.67 ± 19.85	355.83 ± 6.24	350.17 ± 5.06	322.33 ± 14.64	300.75 ± 13.59^*^	323.5 ± 17.19
Shannon	4.21 ± 0.07	3.98 ± 0.07^*^	4.12 ± 0.08	4.05 ± 0.09	3.93 ± 0.11	3.83 ± 0.14^*^	3.9 ± 0.1^*^

Values represent the mean ± SEM (n=4-6). *SEM* standard error of the mean

*p < 0.05 when compared with control, Dunnett's *t* test was used for analysis

### Severe burn disrupted intestinal microbiota

**Fig. 6:**
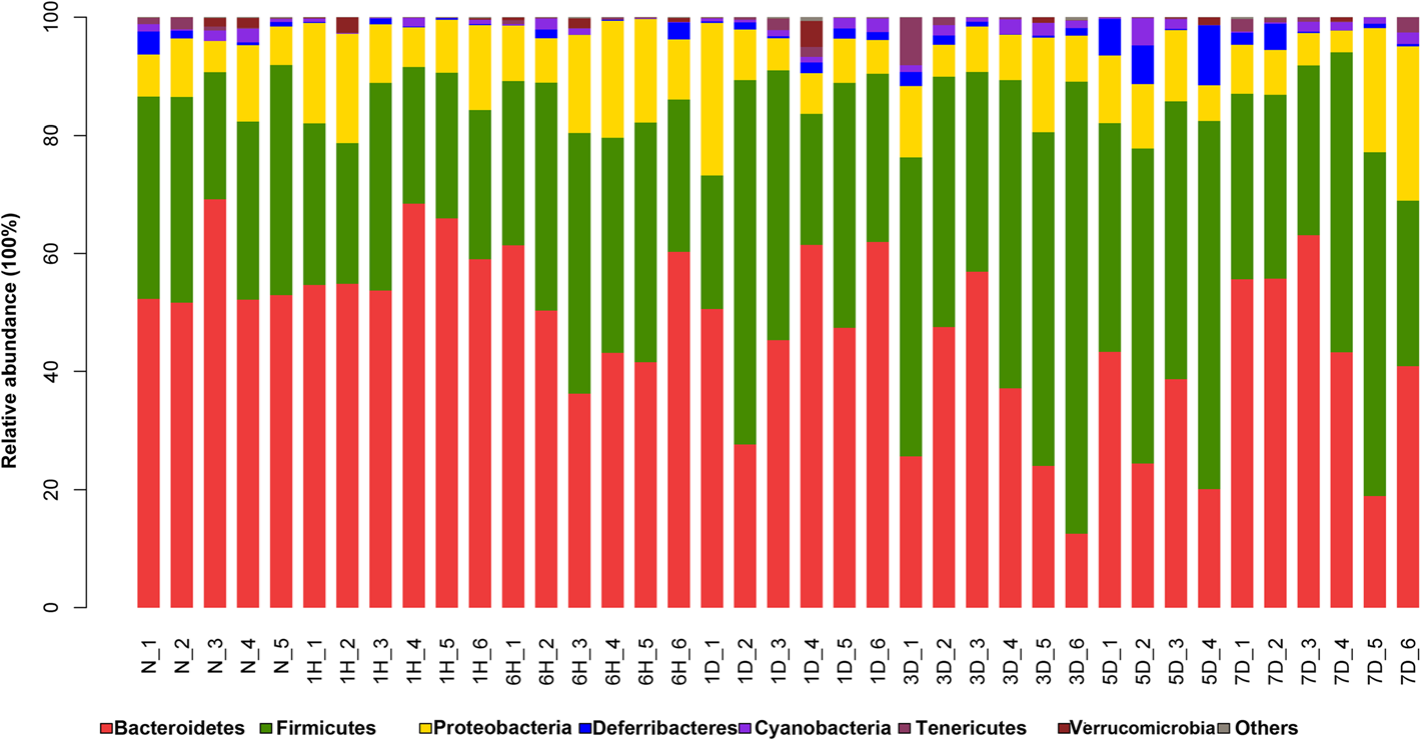
Severe burn disrupted intestinal microbiota. A bar plot about the relative abundances of bacteria at phylum level in each sample. The *X*-axis is grouping information, and the *Y*-axis represents relative abundance of specific species on Phylum level. The relative abundance of *Bacteroidetes* was reduced, whereas the abundance of *Firmicutes* was elevated. Different color represents different species, as indicated. N stands for control group, 1H, 6H stands for 1-h, 6-h post-burn group, 1D, 3D, 5D, 7D stands for 1-day, 3-day, 5-day, 7-day post-burn group

To further understand the alterations of intestinal microbiota after severe burn injury, we next analyzed the intestinal bacterial composition structure in burned mice. As illustrated in Fig. 6, *Bacteroidetes*, *Firmicutes*, and *Proteobacteria* were the three dominant bacteria on phylum level, accounting for 46.39%, 38.88%, and 10.82% of the total sequences, respectively. As showed in Fig. 6 and Additional file 2: Figure S2, the relative abundance of *Firmicutes*, *Bacteroidetes*, and *Deferribacteres* varied a lot among groups. In detail, *Bacteroidetes* declined in the 3-day and 5-day groups; however, *Firmicutes* and *Deferribacteres* elevated in the 3-day and 5-day groups. These results indicate that severe burn injury can obviously change the structure of intestinal microbiota.

### Supervised comparison of intestinal microbiota

**Fig. 7:**
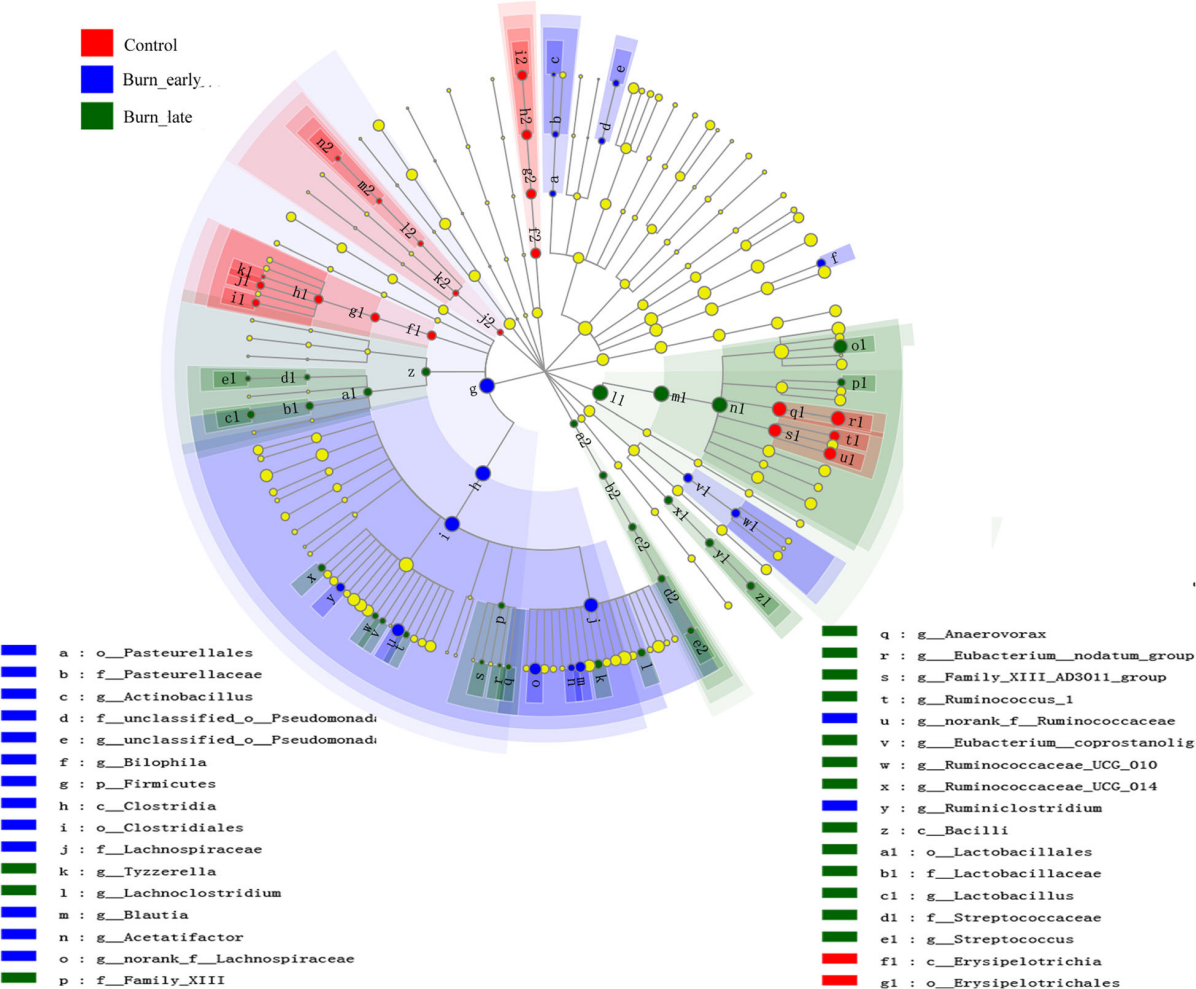
The LefSE analysis of intestinal microbiota. Blue dots symbolize the differential bacteria in control group. Red dots stand for bacteria with significant differences in burn_early group. Green dots represent bacteria that were dramatically modified in burn_late group. The species without apparent variation are represented by yellow dots. The circle is segmented into five layers, respectively symbolizing phylum, class, order, family, and genus levels from the inside out

Based on the community analysis of each sample, we further re-divided these 40 samples into three groups, i.e., control group (N-1 to N-5), burn_early group (1H-1 to 1H-6, 6H-1 to 6H-6, 1D-1 to 1D-6), and burn_late group (3D-1 to 3D-6, 5D-1 to 5D-4, 7D-1 to 7D-6), attempting to highlight the species with different abundance among groups. The results from the principal coordinate analysis (PcoA) [23] showed the considerable discrepancies in microbial composition at OTU level among the three groups (Additional file 3: Figure S3). Furthermore, we analyzed the compositional differences of intestinal microbiota by utilizing linear discriminant analysis effect size, which is a traditional method for bacterial differential analysis [24]. As indicated in Fig. 7, we discovered that *g_Tyzzerella*, *g_Lachnoclostridium*, *g_Anaerovorax*, *g_Ruinococcus_1*, *g_Lactobacillus*, *f_Lactobacillaceae*, and other 10 species had statistical differences in control group. *c_Erysipelotrichia* and *o_Erysipelotrichales* were the discrepant species in the burn_early group. *c_Clostridia*, *g_Bilophila*, *g_Actinobacillus*, *g_Pasteurellaceae*, and other 11 species were the noticeably varied bacteria in the burn_late group. These results indicate that severe burn is capable of disrupting intestinal microbiota, especially during the late stage of burn injury.

### Potential biomarkers analysis

**Fig. 8:**
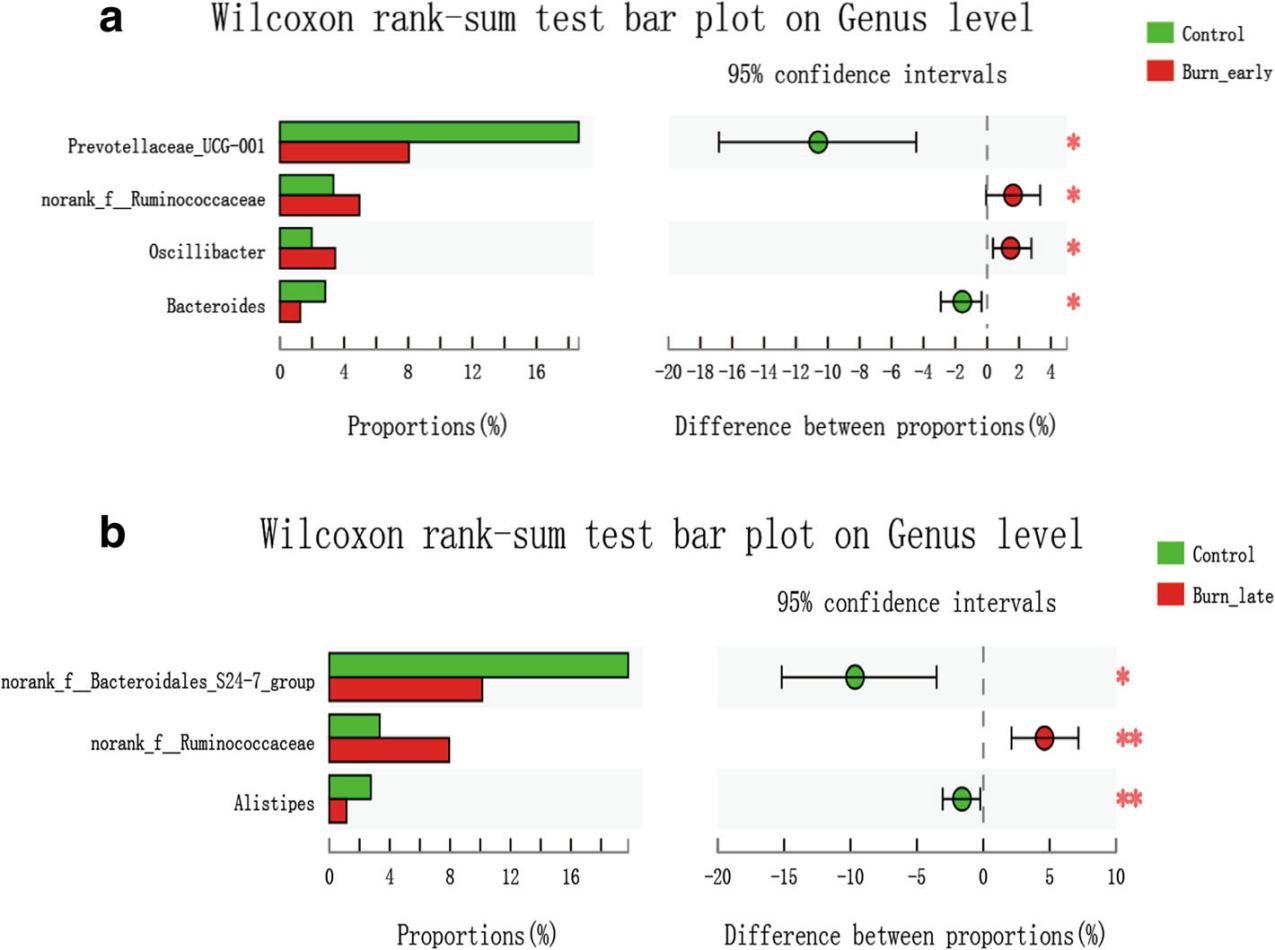
Wilcoxon rank sum test analysis. **a**, **b** The differential bacteria in burn_early group and burn_late group respectively. The *X*-axis is the percentage value of abundance, and the *Y*-axis represents the name of specific species at various classification levels. Control *n*=5; burn_early *n*=18; burn_late *n*=17. *p < 0.05, **p < 0.01 when compared with control group

Based on the LefSE analysis, we further utilized the Wilcoxon rank sum test to figure out the potential biomarkers in burned mice. As shown in Fig. 8a, the specific differential bacteria in the burn_early group were *norank_Ruminococcaceae* (*p* = 0.02), *prevotellaceae_UCG-001* (*p* = 0.012), *Oscillibacter* (*p* = 0.03), and *Bacteroides* (*p* = 0.02). However, as illustrated in Fig. 8b, the specific differential bacteria in the burn_late group were *norank_Ruminococcaceae* (*p* = 0.004), *Alistipes* (*p* = 0.008), and *norank_Bacteroidales_S24-7_group* (*p* = 0.01). Thus, these results indicate that these remarkably altered bacteria species may be considered as the potential biomarkers following severe burn injury.

### Correlations between intestinal microbial community and environmental factors in severe burn

**Fig. 9:**
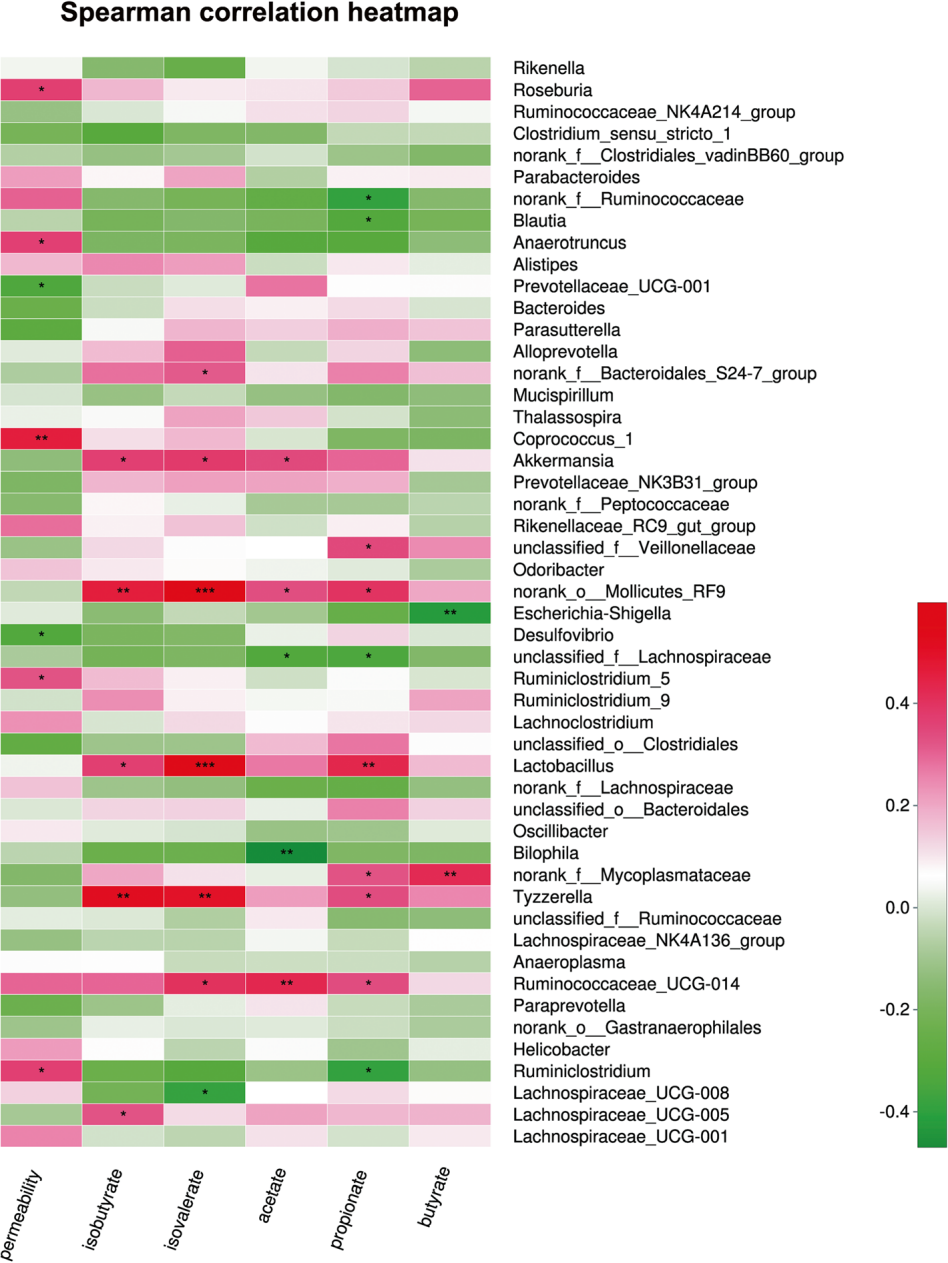
Correlations between intestinal microbial community and environmental factors. The *X*-axis is the environmental factors including intestinal permeability, acetate, propionate, butyrate, isobutyrate, and isovalerate, and the *Y*-axis represents the specific species at genus level. Spearman correlation coefficients are represented by color ranging from blue, negative correlation (− 0.4), to red, positive correlation (0.4). Significant correlations are noted by *p < 0.05, **p < 0.01, ***p < 0.001, respectively

To address the mutual relationship between specific bacteria species (top 50 species in abundance) and environmental factors, we employed the Spearman correlation analysis in this study. In this part, the SCFAs’ contents and intestinal permeability were regarded as environmental factors. As shown in Fig. 9, the intestinal permeability was positively correlated with *Coprococcus_1* (*R* = 0.459), *Anaerotruncus* (*R* = 0.365), *Roseburia* (*R* = 0.37), *Ruminiclostridium_5* (*R* = 0.327), and *Ruminiclostridium* (*R* = 0.367), but negatively correlated with *Desulfovibrio* (*R* = − 0.322) and *Prevotellaceae_UCG-001* (*R* = − 0.331). Among the SCFAs, isobutyric acid was positively correlated with *Lachnospiraceae_UCG-005* (*R* = 0.326), *Tyzzerella* (*R* = 0.516), *Lactobacillus* (*R* = 0.37), *norank_o_Mollicutes_RF9* (*R* = 0.461), and *Akkermansia* (*R* = 0.367). Isovaleric acid was positively correlated with *Ruminococcaceae_UCG-014* (*R* = 0.405), *Tyzzerella* (*R* = 0.5), *Lactobacillus* (*R* = 0.564), *norank_o_Mollicutes_RF9* (*R* = 0.598), *Akkermansia* (*R* = 0.382), and *norank_f_Bacteroidales_S24-7_group* (*R* = 0.316), but negatively correlated with *Lachnospiraceae_UCG-008* (*R* = − 0.387). Butyrate was positively correlated with *norank_f_Mycoplasmataceae* (*R* = 0.422), but negatively correlated with *Escherichia-Shigella* (*R* = − 0.424). Acetate was negatively correlated with *unclassified_f_Lchnospiraceae* (*R* = − 0.321) and *Bilophila* (*R* = − 0.47), but positively correlated with *Ruminocaccaceae_UCG-014* (*R* = 0.431), *norank_o_Mollicutes_RF9* (*R* = 0.336), and *Akkermansia* (*R* = 0.345). Propionate is negatively correlated with *Blautia* (*R* = − 0.314), *Ruminiclostridium* (*R* = − 0.392) *unclassified_f_Lachnospiraceae* (*R* = − 0.34), and *norank_f_Ruminococcaceae* (*R* = − 0.396), but positively correlated with *Ruminococcaceae_UCG-014* (*R* = 0.339), *Tyzzerella* (*R* = 0.339), *norank_f_Mycoplasmataceae* (*R* = 0.327), *Lactobacillus* (*R* = 0.435), *norank_o_Mollicutes_RF9* (*R* = 0.399), and *unclassified_f_Veillonellaceae* (*R* = 0.347). In brief, *norank_o_Mollicutes_RF9*, *Akkermansia*, *Lactobacillus*, *Tyzzerella*, and *Ruminococcaceae_UCG-014* were the principal species correlated with SCFAs including acetate, propionate, butyrate, isobutyrate, and isovalerate. These results indicate that the variation of intestinal microbiota composition is closely associated with some environmental factors in severe burn injury.

## Discussion

The main original discovery of this study is that severe burn injury disrupts intestinal barrier through affecting the expressions and morphology of tight junction proteins. Meanwhile, the microbial diversity and composition are significantly changed by severe burn injury. Furthermore, severe burn injury also decreases SCFAs’ concentrations and changes inflammatory cytokines’ expressions. These experimental data provides a new insight into the associations among the severe burn-induced intestinal barrier dysfunction, microbiota dysbiosis, inflammatory cytokines, and SCFAs.

It has been well established that severe surgical illness, such as shock, trauma, and burn injury, can destroy intestinal epithelial barrier, resulting in the leakage of bacteria and bacterial metabolites from the intestinal lumen into the mucosa or circulatory system [5, 6, 25]. Based on this study, we identify again that severe burn injury causes intestinal barrier dysfunction, which presents as the increased intestinal permeability, altered tight junction proteins’ expressions, and disrupted ZO-1 morphology. These results are in consistent with the previous studies which show that the burn-induced intestinal barrier disruption is characterized by augmented pare-cellular permeability and altered tight junction proteins’ expressions or morphology [26–28]. Thus, there is no doubt that severe burn injury can disrupt the integrity of intestinal epithelial barrier.

Given that severe burn induces intestinal barrier defect, however, the underlying mechanisms are still unclear. Actually, early intestinal disruption caused by burn injury is mainly manifest as a series of pathophysiological events such as inflammation, decreased SCFAs, and intestinal microbiota dysbiosis [29, 30]. It has been reported that the altered intestinal microbiota contributed to augmented intestinal para-cellular permeability [31]. In this study, the intestinal permeability is positively correlated with *Coprococcus_1*, *Anaerotruncus*, *Roseburia*, *Ruminiclostridium_5*, and *Ruminiclostridium*, but negatively associated with *Desulfovibrio* and *Prevotellaceae_UCG-001*. Thus, the intestinal microbiota dysbiosis may contribute to the occurrence of intestinal barrier disruption induced by severe burn injury. In addition, SCFAs have been reported to be able to enhance intestinal barrier [32]. By the way, SCFAs show protective and stimulative effects on intestinal barrier through inhibiting autophagy and NLRP3 (NLR family, pyrin domain containing protein 3) inflammasome [33]. Therefore, the reduced SCFAs contents observed in present study may also contribute to the severe burn-induced intestinal barrier dysfunction. Furthermore, this study also reveals that the pro-inflammatory cytokines including IL-1β and IL-6 are elevated after severe burn injury, which is consistent with the previous reports [34–36]. It is well documented that both IL-1β and IL-6 can disrupt intestinal barrier [37]. Hence, it is rational to speculate that inflammation, intestinal microbiota dysbiosis, and decreased SCFAs contribute to intestinal barrier disruption caused by severe burn injury.

Here, we also show the community-wide changes of intestinal microbiota in severely burned mice. The detailed compositional alterations in the intestinal microbiota following burn injury were investigated at different bacterial taxonomic levels. On phylum level, the predominant phyla were *Bacteroidetes*, *Firmicutes*, and *Proteobacteria*. After severe burn injury, the proportion of *Bacteroidetes* was decreased, whereas the proportions of *Firmicutes*, *Proteobacteria*, and *Deferribacteres* were increased, resulting in an elevation in *Firmicutes*/*Proteobacteria* ratio. On genus level, the genera *Mollicutes_RF9_g_norank*, *Candidatus_Saccharimonas*, *Butyricimonas*, *Ruminococcaceae_UCG_010*, and *Ruminiclostridium_6* were elevated after severe burn. Thus, it is suggested that the decreased microbial diversity estimated by the Shannon Index in present study may be associated with burn-induced inflammatory response, because decreased diversity of intestinal microbial community has been reported in intestinal inflammation [38].

The composition of individual gut microbiota is believed to be influenced by various factors, such as host genetic elements, immune responses, antimicrobial substances, microbial interactions, and environmental factors [39]. Therefore, based on the observed shifts in bacterial community after burn injury, we further investigated the potential changes of intestinal environmental and fermentative factors. The contents of luminal SCFAs including acetate, propionate, butyrate, isobutyrate, and isovalerate were significantly decreased after severe burn. Similarly, other investigators have also shown that burn injury decreases butyrate contents in the colon of burned mice [40]. Although the mechanism involved in burn decreased SCFAs contents is not clear, it has been documented that decrease in *Firmicutes*/*Proteobacteria* ratio leads to increased SCFAs contents [41]. Thus, it is rational to speculate that an elevation in *Firmicutes*/*Proteobacteria* ratio observed in this study may be an important cause of the reduced SCFAs contents after severe burn injury.

This study also demonstrates the relationship between specific species of intestinal microbial community and variations of SCFAs contents following severe burn injury. The principal species correlated with SCFAs contents after severe burn injury were *Lactobacillus*, *Akkermansia*, *Tyzzerella*, *norank_o_Mollicutes_RF9*, and *Ruminococcaceae_UCG-014*. It has been documented that *Ruminococcaceae* can produce acetate [42], and *Lactobacillus* is able to produce lactate which can be utilized by the bacteria belonging to the *Clostridial* cluster XIVa to produce butyrate [43]. Besides, the contents of acetate, butyrate, and propionate have been reported to be positively correlated with lactic acid bacteria such as *Bacillales*, *Sporolactobacillus*, and *Lactobacillus* [35]. Thus, our present data, which shows the correlations between bacteria species and SCFAs contents, may provide some implications for understanding the association between intestinal microbiota composition and SCFAs production after severe burn injury.

## Conclusions

In conclusion, our present study reveals the variation tendencies of intestinal barrier, microbiota, SCFAs contents, and inflammatory cytokines expressions after severe burn injury. However, the exact relationship between intestinal microbiota dysbiosis and barrier dysfunction following severe burn needs further investigations. A deeper understanding of host-microbial crosstalk might be helpful to the development of novel alternative strategies as well as approaches to restore intestinal barrier after severe burn injury.

## Additional files

Additional file 1: **Figure S1.** Rarefaction curve of the fecal samples (97% similarity). Repeated samples of operational taxonomic unit (OTU) subsets were used to evaluate whether further sampling would likely yield additional taxa, as indicated by whether the curve has reached a plateau value. N stands for control group, 1H, 6H stands for 1-h, 6-h post-burn group, 1D, 3D, 5D, 7D stands for 1-day, 3-day, 5-day, 7-day post-burn group. (TIF 1401 kb)

Additional file 2: **Figure S2.** Abundance alteration of specific bacteria on Phylum level. Abundance of each bacteria on Phylum level was assessed in this experiment. The *X*-axis is grouping information, and the *Y*-axis represents the abundance of species on Phylum level. After burn injury, the abundance of *Bacteroidetes* decreased significantly at 3 days and 5 days; however, abundance of *Firmicutes* and *Deferribacteres* increased at 3 days and 5 days. Data represent the mean ± standard error of the mean (SEM) (*n* = 4–6). ^*^p < 0.05 compared with control, Dunnett's *t* test was used for analysis. Ctrl stands for control group, 1H, 6H stands for 1-h, 6-h post-burn group, 1D, 3D, 5D, 7D stands for 1-day, 3-day, 5-day, 7-day post-burn group. (TIF 254 kb)

Additional file 3: **Figure S3.** Principal coordinates analysis (PCoA) using weighted unifrac. The green dots indicate the distributions of samples in the control group. The red triangles stand for samples in burn_early group. The blue quadrangles represent the specimens in the burn_late group. *OTU* operational taxonomic unit. (TIF 244 kb)

## Abbreviations

FITC-dextran: Fluorescein isothiocyanate-labeled dextran
HS-GC/MS: Headspace gas chromatography-mass spectrometry
OTU: Operational taxonomic unit
PBS: Phosphate buffer saline
PCoA: Principal coordinates analysis
PDVF: Polyvinylidene difluoride
SCFA: Short-chain fatty acid
SIM: Single ion monitoring
TBSA: Total body surface area
ZO-1: Zonula occludens-1
