# Supplementary figures and images for: Severe burn injury alters intestinal microbiota composition and impairs intestinal barrier in mice
Source: Burns Trauma. 2019 Jul 4;7:20. doi: 10.1186/s41038-019-0156-1 (PMC6610819; doi:10.1186/s41038-019-0156-1)

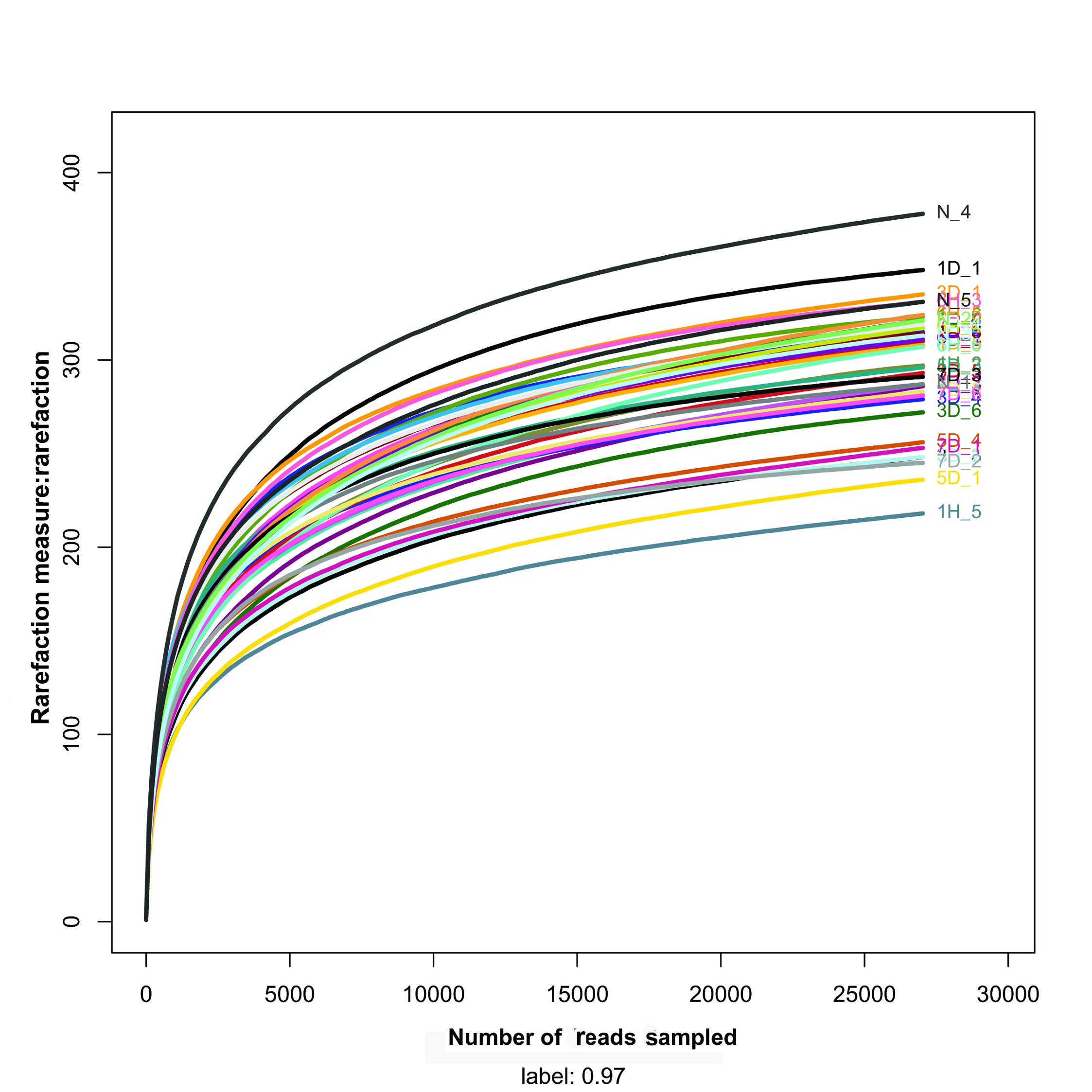

Supplement: Supplementary file 1 — Figure S1. Rarefaction curve of the fecal samples (97% similarity). Repeated samples of operational taxonomic unit (OTU) subsets were used to evaluate whether further sampling would likely yield additional taxa, as indicated by whether the curve has reached a plateau value. N stands for control group, 1H, 6H stands for 1-h, 6-h post-burn group, 1D, 3D, 5D, 7D stands for 1-day, 3-day, 5-day, 7-day post-burn group. (TIF 1401 kb) [file 41038_2019_156_MOESM1_ESM.tif]

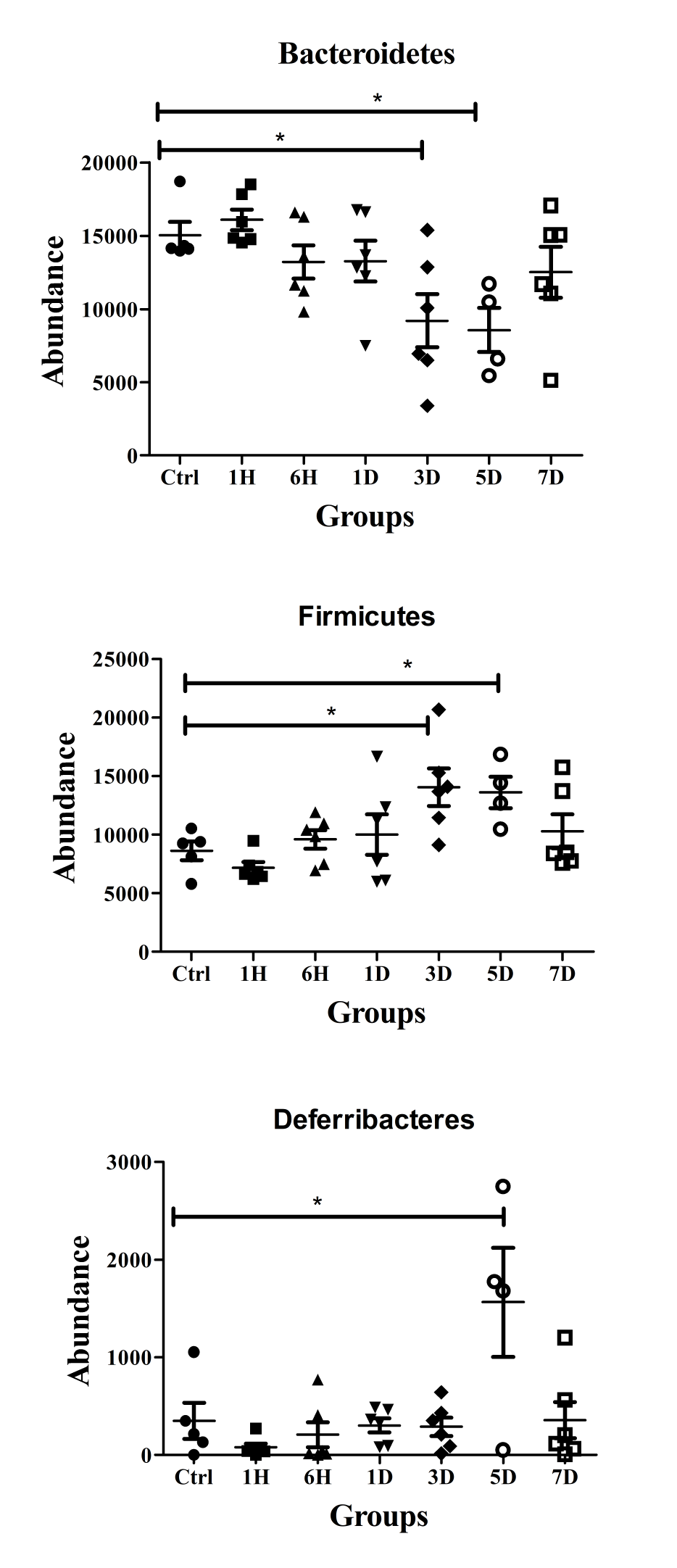

Supplement: Supplementary file 2 — Figure S2. Abundance alteration of specific bacteria on Phylum level. Abundance of each bacteria on Phylum level was assessed in this experiment. The X-axis is grouping information, and the Y-axis represents the abundance of species on Phylum level. After burn injury, the abundance of Bacteroidetes decreased significantly at 3 days and 5 days; however, abundance of Firmicutes and Deferribacteres increased at 3 days and 5 days. Data represent the mean ± standard error of the mean (SEM) (n = 4–6). *p < 0.05 compared with control, Dunnett's t test was used for analysis. Ctrl stands for control group, 1H, 6H stands for 1-h, 6-h post-burn group, 1D, 3D, 5D, 7D stands for 1-day, 3-day, 5-day, 7-day post-burn group. (TIF 254 kb) [file 41038_2019_156_MOESM2_ESM.tif]

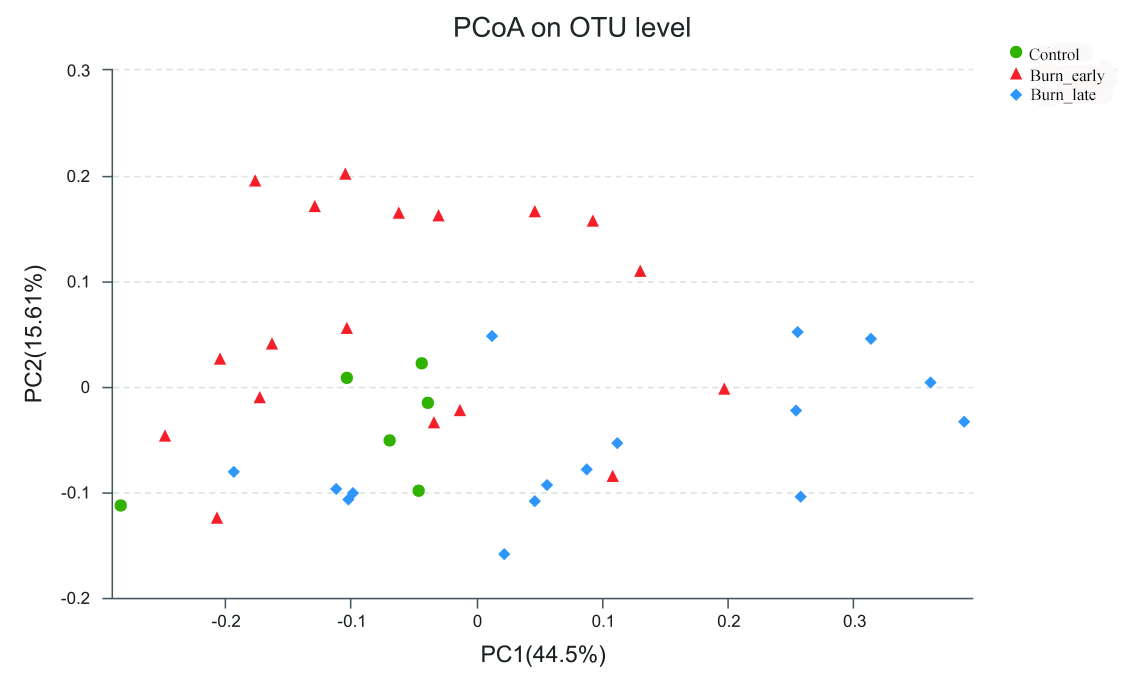

Supplement: Supplementary file 3 — Figure S3. Principal coordinates analysis (PCoA) using weighted unifrac. The green dots indicate the distributions of samples in the control group. The red triangles stand for samples in burn_early group. The blue quadrangles represent the specimens in the burn_late group. OTU operational taxonomic unit. (TIF 244 kb) [file 41038_2019_156_MOESM3_ESM.tif]
